# What is the impact of inflammasome mechanisms on male infertility?

**DOI:** 10.55730/1300-0144.5631

**Published:** 2023-03-07

**Authors:** Eser ÖRDEK, Bülent KATI, İsmail KOYUNCU, Mehmet DEMİR, İsmail YAĞMUR, Eyyüp Sabri PELİT, Halil ÇİFTÇİ, Ercan YENİ

**Affiliations:** 1 Department of Urology, Faculty of Medicine, Harran University, Şanlıurfa, Turkey; 2 Department of Biochemistry, Faculty of Medicine, Harran University, Şanlıurfa, Turkey; 3 Department of Urology, Ankara State Hospital, Ankara, Turkey

**Keywords:** Infertility, azoospermia, varicocele, NLRP3, inflammasome

## Abstract

**Background/aim:**

Mechanisms to explain inflammation in male infertility of unknown cause are still being investigated. The inflammasome is a key regulator of innate immunity in the inflammatory response to infections. Our study aims to investigate the effects of varicocele on infertility, its relationship with antioxidant and inflammasome mechanisms, and how it could be guided in azoospermic or nonazoospermic patients.

**Materials and methods:**

A cross-sectional cohort study was conducted at the department of urology in our university hospital. Eighty-eight randomly selected men aged 20–45 admitted to our hospital because of infertility between September 2019 and July 2020 were included in the study. Patients were divided into four equal groups according to their clinical status, those with/without azoospermia and with/without varicocele. Blood and semen samples were taken from the patients. NOD-like receptor pyrin domain-containing 3 (NLRP3) and interleukin-1 beta (IL1β) and total antioxidant status (TAS), total oxidant status (TOS), and oxidative stress index (OSI) levels were measured in serum and semen, and the groups were compared statistically.

**Results:**

Serum and semen NLRP3, IL1β, TAS, TOS, and OSI values of the patients with varicocele or azoospermia were significantly higher than those without either varicocele or azoospermia (p < 0.05). The oxidative stress markers TAS, TOS, and OSI values were significantly higher in the other groups than those without azoospermia and varicocele (p < 0.05).

**Conclusion:**

Inflammasome mechanisms, such as NLRP3 and IL1-β molecules, may provide additional benefit in evaluating the need and benefit of surgical or medical treatment in infertility with and without vascular pathology and with and without azoospermia.

## 1. Introduction

Infertility is the inability to have children despite one year of regular sexual intercourse without contraception [1]. This medical condition can lead to social and psychological problems, affecting 8%–12% of couples of reproductive ages and over 186 million people worldwide [2]. Even if new studies conducted in recent years have tried to clarify the causes of male infertility, there are still many unknowns related to this condition. High levels of specific cytokines present during persistent infection/inflammation in the male genital tract can increase peroxidation [3] process and subsequently affect sperm function with the development of infertility, although many studies have shown improvement in sperm parameters in semen samples after varicocele repair. The effect of varicocele repair on improving spermatogenesis in men with nonobstructive azoospermia (NOA) has still not been fully elucidated [4]. Varicocele also is the most common etiological factor associated with male infertility. It is a vascular disorder of the testis defined as the dilated veins of the pampiniform plexus. There is also important evidence in the literature that varicocele harms testicular and sperm function by inflammatory mechanisms [5]. Inflammasome is a multiprotein complex in the cell’s cytoplasm, and uric acid crystals are aggregated in response to various stimuli as a distress signal such as reactive oxygen species (ROS), ATP, free fatty acid (FFA), high mobility group box1 (HMGB1), heat shock proteins, and pathogens. Activation of the inflammasome results in the maturation of inflammatory cytokines such as IL-1β and IL-18 [6].

Inflammasomes are key regulators of innate immunity involved in the inflammatory response to infections and disease, emerging via the activation of caspase-1 and the processing of the inflammatory cytokines interleukin (IL) 1β and IL18 [7]. Among the different types of inflammation (nucleotide binding and oligomerization domain [NOD]-like receptor family pyrin domain 1 [NLRP1], NLRP2, NLRP3, and AIM (absent in melanoma)), there are many studies on the role of the NOD-like receptor family, pyrin-containing 3 (NALP3) inflammasome, particularly experimental infertility studies on its groups NLRP3 and IL1β [8]. We planned this prospective randomized study to investigate this inflammasomal (NLRP3, IL1) mechanism in men with varicocele and/or azoospermia because it represents one of the important etiological causes of male infertility. We also aimed to determine the degree of inflammation via tests (total antioxidant level [TAS], total oxidant level [TOS], oxidative stress index [OSI]).

## 2. Materials and methods

Approval was received from the local ethics committee of our university hospital for a cross-sectional cohort study conducted at the department of urology. Male patients who applied to our university hospital, faculty of medicine, and department of urology-andrology outpatient clinic for infertility between September 2019 and July 2020 were recruited. After giving detailed information about the research, we asked them whether they would participate in the study. A total of 88 men—aged 20–45 years—who could not have children despite regular and unprotected sexual intercourse for at least 1 year (6 months for > 35 years old) were included in the study. Patients with significant leukocytes (>1 million/mL) in semen; individuals over the age of 45 years; patients with active epididymo-orchitis or urinary tract infection; patients with undescended testis, testicular torsion, testicular trauma, or tumor history; patients taking pentoxifylline, vitamins, and patients with chronic smoking were excluded from the study. Azoospermic patients with abnormal karyotype analysis, patients with y microdeletion, and patients with abnormal prolactin, FSH, and testosterone levels were also excluded to reduce the study’s limitations. Only diagnosed patients with NOA were included in the azoospermic study group.

Semen samples were taken after at least 72 h of the last ejaculation, and random venous blood samples were taken between 08.00 and 11.00 AM during the fasting period. Seminal plasmas were taken into 2-mL Eppendorf tubes after liquefaction, centrifuged at 3000 rpm for 15 min, and then stored at −80 °C. Similarly, venous (antecubital vein) blood samples were taken from the participants to study NLRP3 (BT lab. China™) and IL1-β (BT lab. China™); oxidative stress parameters, including TOS and TAS, were measured (Rel Assay Diagnostics Turkey™) and the OSI was calculated. Blood samples taken into biochemistry tubes were kept at room temperature for 30 min to coagulate, and serum samples were separated by centrifugation at 4000 rpm for 10 min. Serum samples obtained were stored at −80 °C until analysis. Patients with at least grade 2–3 clinical varicocele were named Varicocele (+). Patients without clinical varicocele were named Varicocele (−).

Patients with at least grade 2–3 clinical varicocele were named Varicocele (+). Patients without clinical varicocele were named Varicocele (−). Patients with nonobstructive azoospermia in at least two different spermiogram measurements and evaluations were named Azoospermia (+), and patients with at least 5 million/mL sperm count were named Azoospermia (−).

As a result of the evaluations, diagnoses of azoospermia (−)/varicocele (−), azoospermia (−)/varicocele (+), azoospermia (+)/varicocele (−), and azoospermia (+)/varicocele (+) were established, with each group consisting of 22 individuals. Four separate working groups were formed, labeled Group I, Group II, Group III, and Group IV, respectively.

After the sperm and blood collection procedure, frozen samples were thawed gradually just before analysis. NLRP3, IL1β, TAS, TOS, and OSI were studied in the serum and sperm samples of each patient in the laboratory of the biochemistry department of our university, faculty of medicine research and application hospital.

### 2.1. Determination of NLRP3, interleukin-1 beta, TAS, and TOS

NLRP3 and IL1β levels were studied with the competitive enzyme immunoassay, an immunochemical method. A commercial ELISA kit (NLRP3 ELISA Lot: 202006/Interleukin-1 Beta ELISA LOT:202006) was used. This kit is an enzyme-linked immunosorbent test (ELISA). The plate is precoated with a human NLRP3 antibody. NLRP3 in the sample is added and binds to antibodies coated on the wells. A biotinylated human NLRP3 antibody is added and binds to NLRP3, and streptavidin-HRP is added and binds to the biotinylated NLRP3 antibody. After incubation, unbound streptavidin-HRP is washed off during a wash step. The substrate solution is added, and the color develops proportionately to the amount of human NLRP3. The reaction is terminated by adding the acidic stop solution, and the absorbance is measured at 450 nm. Interleukin-1 beta was also studied with similarly labeled ELISA kits to measure serum and semen levels.

The samples’ total oxidant status (TOS) levels were measured using Rel Assay brand commercial kits. The measurement was made using the colorimetric method, which is based on the cumulative oxidation of the oxidant molecules contained in the samples to ferrous ion ferric ion, as stated in the working principle of the test. Results are expressed as μmol H2O2 (hydrogen peroxide) equivalent/L. (Rel Assay® Diagnostics kits: Mega Tip, OK20115O Gaziantep, Turkey).

The samples’ total antioxidant status (TAS) levels were measured using Rel Assay brand commercial kits. The measurement was based on the cumulative oxidation of ferrous ion to ferric ion by the oxidant molecules contained in the samples, as stated in the working principle of the test, and a colorimetric method was used. Results were expressed as μmol H2O2 equivalent/L.

The oxidative stress index (OSI), which is shown as an indicator of oxidative stress, is expressed as a percentage of the ratio of total oxidative status/level (TOS) to total antioxidant status/level (TAS). When calculating the samples’ oxidative stress index (OSI), the TAS levels are multiplied by 10 to equalize the TOS levels and the units. Results are expressed as arbitrary units (AU).

### 2.2. Statistical analysis

In the descriptive statistics of the data, the mean, standard deviation, median, and lowest and highest values were used. The distribution of variables was measured with the Kolmogorov–Smirnov test. Kruskal–Wallis and Mann–Whitney U tests were used to analyze independent quantitative data. SPSS 26.0 program software (IBM Corp., Armonk, NY, USA) was used in the analyses.

## 3. Results

There was no significant difference between the mean age of the patients and the findings and the average of biochemical serum and sperm results.

In our study, like NLRP3, IL1-semen and serum values were studied in all groups, and serum values were significantly higher in patients with varicocele alone (Group 2–4) compared to patients without azoospermia and varicocele (Group 1) regardless of the azoospermia status. However, it was significantly higher in patients without varicocele (Group 3) compared to Group 1. IL1-measured in semen values were significantly higher in patients with varicocele alone (Group 2–4) compared to patients without azoospermia and varicocele (Group 1) regardless of the azoospermia status, while groups with or without azoospermia had varicocele (Group 3–4).), there was no significant difference compared to the group without azoospermic varicocele (Group 2). Thus, we saw that the IL1-β value in semen increased significantly with varicocele status.

The first purpose in this study, in the groups evaluated and created according to azoospermic patients. The standard effect size was determined as 0.84 with 5 % error margin and 80% power analysis.

### 3.1. Analysis of serum parameters of patient groups

Serum IL-1 β in group IV, group III and group II were significantly higher than in group I (p = 0.041). Serum NLRP3 value in group IV was significantly higher than group I and group III (p = 0.022). Serum TOS value was significantly higher in group IV, group III, and group II than in group I (p < 0.001). Serum TAS value was significantly higher in group IV, group III, and group II than in group I (p < 0.001). Serum OSI value was significantly higher in group IV, group III, and group II than in group I (p < 0.001). Serum OSI values in group IV and group III were significantly higher than in group II (p < 0.001) (Table).

### 3.2. Analysis of semen parameters of patient groups

Semen IL-1 β value was significantly higher (p < 0.001) in group IV, group III, and group II than in group I. Semen IL-1 β value in group IV was significantly higher than in group III (p < 0.001). Semen IL-1 βvalue did not differ significantly between group III and group II (p > 0.05). Semen NLRP3 value in group IV was significantly higher than group I and group III (p = 0.002). The sem en TOS value was significantly higher in group IV, group III, and group II than in group I (p < 0.001). The semen TOS value was significantly higher in group IV than in groups II and III (p = 0.000). The semen TAS value in group IV, group III, and group II were significantly higher than in group I (p < 0.001). Semen OSI values in group IV, group III and group II were significantly higher than in group I (p < 0.001). The semen OSI value in group IV was significantly higher than group II (p < 0.001) (Table).

## 4. Discussion

Inflammatory periods accused of unknown infertility and inflammasome mechanisms are being questioned more and more day by day. There are a variety of causes of male infertility, from genetic mutations to lifestyle choices, medical illnesses or inflammation to varicocele and medications [9]. Several known inflammasome platforms exist, such as the nucleotide binding and oligomerization domain-like receptor family pyrin domain 1 (NLRP1), NLRP3, AIM2, and NLRC4 inflammation [10]. The oxidative stress index of the samples is calculated as the ratio of the total oxidant levels of the samples to the total antioxidant levels and expressed as a percentage. In this way, many studies on infertility are available in the literature [11,12]. It is a limitation for us that they are affected by any unknown condition while working with antioxidant markers in the systemic blood.

In the semen analysis of male patients presenting with infertility, a marked deterioration of 90% is usually observed in the parameters [13]. Especially in male infertility of unknown etiology, increasing studies at the cellular and molecular level to reveal the pathophysiology of the problem is important in revealing the unknown aspects of infertility in the future. One of the important issues studied is free oxygen radicals and semen parameter disorders, and it is understood that free oxygen radicals (FOR) play an important role in infertility [14]. Oxidative stress resulting from the imbalance between antioxidants and free oxygen radicals in the body causes infertility by damaging sperm [15].

Varicocele is the most common cause of male infertility with a multifactorial etiology. In men presenting with infertility, varicocele should be carefully evaluated during a physical examination, and radiological methods should be used if necessary. The incidence of varicocele is 4.4%–22.6% in the general population, 21%–41% in men with primary infertility, and 75%–81% in men with secondary infertility [16]. It is assumed that varicocele, which has a large share in secondary infertility, does this through various mechanisms. In varicocele disease, temperature increase stress caused by venous pooling causes undesirable adverse effects such as spermatogenesis disorder on testicular tissue, increased production of reactive oxygen species (ROS) and apoptosis [17]. Inflammation is a characteristic pathological event in the testicular tissue following varicocele. Studies show that varicocele stimulates the release of proinflammatory and inflammatory cytokines such as interleukin-1 (IL-1), IL-6, IL-8, and tumor necrosis factor-alpha (TNF-α) [18]. Studies have proven that antioxidant molecules protect spermatozoa cells from abnormal spermatozoa that produce free oxygen radicals, remove free oxygen radicals, which are products of leukocytes, prevent DNA fragmentation, increase sperm quality in smoking individuals, and prevent premature sperm maturation [19].

Moretti et al. investigated whether semen features in different clinical infertility diagnoses are associated with PMN elastase, IL-6, IL-8, IL-1b, and TNFa levels detected in seminal plasma, and idiopathic infertility (group I), infectious varicocele (group II), varicocele (group III), infections (group IV), and controls (group V) on 68 patients. As a result of the study, changes in semen quality and normal inflammatory mediator levels were observed in patients with idiopathic infertility. They concluded that genitourinary infection and varicocele caused an inflammatory effect that could play a detrimental role in spermatogenesis [18].

Recent studies have shown that inflammatory activation and, consequently, increase of pro-inflammatory cytokines in seminal plasma lead to impaired spermatogenesis in men [20,21]. Promoting leukocyte activation may be a key player in the relationship between infertility and inflammation [22,23].

The leukocyte count in semen analysis may not always give the appearance of true inflammation because a wide range of cells can respond, which is considered the source of inflammatory mediators. This provides the advantage of the amount of NLRP3 as a biomarker for inflammatory processes, and NLRP3’s being the target molecule is important for improvement in sperm cell quantity and quality [24].

In our study, we aimed to bring a new perspective to the inflammatory and antioxidant hypothesis in the etiology of infertility and show the possibility of bringing new treatment targets to the agenda in different infertility patients. In this context, we discussed the NLRP3 inflammation, a multiprotein complex that initiates IL-1β–mediated inflammatory responses, and its relationship with male infertility. Among the objective parameters we examined with the data we obtained from our study, we investigated the effect of serum and sperm NLRP3, IL-1β, TAS, TOS, and OSI markers determining intracellular oxidative stress in infertile men with azoospermia and varicocele. While doing this study, we tried to understand the factors determining this cellular stress by grouping infertile patients with and without azoospermic and clinical varicocele status.

In related studies by Curtin and Erfani et al., varicocele was shown to increase free oxygen radicals in infertile patients and, at the same time, suppress the activity of antioxidant enzymes [25,26]. We found that the oxidative stress markers TAS, TOS, and OSI values of infertile patients in our study were significantly higher in both groups (varicocele + azoospermic/nonazoospermic) in patients with and without azoospermic varicocele compared to patients with varicocele and no azoospermia. This showed us that varicocele alone increases oxidative stress markers in serum and semen values of infertile patients independent of azoospermia status.

In a recent study by Younis et al., the authors tried to determine the seminal plasma level of NLRP3 protein in infertile men using the ELISA method, and the study reported that the average NLRP3 was 5.12 ± 1.18 ng/mL, and this could reflect an inflammatory condition in infertile men [27]. They also studied our study more comprehensively on serum and semen analyses of patients and tried to understand how NLRP3 responds in infertile men depending on varicocele and azoospermia. In our study, NLRP3 serum and semen values were varicocele compared to patients without azoospermia and varicocele. However, no significant increase was found in patients with varicocele without azoospermia compared to patients without varicocele independent of azoospermia. This showed us that besides varicocele, azoospermia significantly increases NLRP3 levels; thus, this proinflammatory molecule gains more importance in azoospermic and patients with varicocele.

In addition, the parallelism of these NLRP3 values in serum and semen analysis showed that it could give us information about this inflammation process even in serum evaluation except in semen. Because the patients we included in the study did not have any known chronic, genetic, or metabolic disorders.

In the study of Baazam et al., in 32 varicocele patients compared to normal patients, interleukin-1b (IL-1b), IL-18, and caspase-1 concentrations in seminal plasma were measured and evaluated by ELISA. The findings of this study indicate that NLRP3 activation occurs in varicocele and may be responsible for the pathological process that occurs in varicocele patients. The details of the NLRP3 inflammatory activation process have not yet been clarified, and whether NLRP3 is caused by the onset of varicocele or is a pathological consequence of this gonadal disease course. They stated that it is not clear [6].

In our study, the NLRP3 value was higher in azoospermic and varicocele patients (Group 4) compared to groups without varicocele (Group 1–3) and was not significantly higher than those with varicocele but without azoospermia (Group 2). We can conclude that it did not have much effect.

Sahin et al. studied the effect of varicocele on the levels of IL-1α and IL-1 β proteins in testicular tissue in an experimental rat model, and it caused testicular damage, especially in 11- and 13-week varicocele-induced rat groups, and IL-1α expression with the progression of varicocele, transiently increased IL-1β expression in Sertoli cells, spermatogonia, primary spermatocytes, spermatids and Leydig cells was observed in Leydig cells in sham-operated rats. They found that IL-1β expression was also increased upon the progression of varicocele, especially in Leydig cells, Sertoli cells, and spermatogonia. As a result, it is stated that IL-1α and IL-1β may be regulators of testicular function, certain pathological conditions such as varicocele cause an increase in the expression of such proinflammatory cytokines, and the increased expression of IL-1α and IL-1β in varicocele may change the balance in favor of inflammatory and immune responses. They argued that it might cause harmful effects on testicular tissue that may cause male infertility [28].

In a recent laboratory study by Antonuccio et al., the roles of selenium and polydeoxyribonucleotide (PDRN) were investigated in an experimental varicocele model in rats, especially considering the role of NLRP3 inflammasome, and the Se-PDRN relationship significantly improved all morphological parameters and significantly increased testosterone levels. They also found that it reduced the expression of NLRP3 inflammasome, caspase-1 and IL-1β. As a result, they thought that NLRP3 inflammasome could be considered an interesting target in varicocele and that Se-PDRN could be a new medical approach supporting surgery [29]. Our clinical study also provides strong evidence that such inflammasomes can identify appropriate antioxidants that can be used to determine the necessity of varicocele surgery and eliminate the potential mechanisms of action that may occur.

As a result, although NLRP3 and its associated IL1-molecule can be used in the inflammatory and regulatory mechanisms to compare or predict the explainable or unexplained mechanisms of infertility, the proportionality and comparability of semen and serum values to each other can increase the usability of these molecules. It would be a promising approach to aim the antiinflammatory or antioxidant drugs to be developed with this study, which makes it possible to make certain classifications and evaluate them while investigating infertility patients, to reduce the formation of inflammatory and oxidant molecules to reduce the pro-inflammatory environment. In addition, determining to what extent the degree of varicocele affects the inflammasome mechanism in patients will guide the treatment decision in infertile varicocele men regarding the surgical requirement.

In the recent study of Kati et al., the relationship between the inflammasome mechanism and testis sperm extraction success was investigated. The authors found that the effects of NLRP3 and IL-1 inflammasomes are significant inflammation markers on mTESE success in azoospermic infertile men [30].

Although this study tried to achieve standardization, the patients’ medical history, chronic known or unknown diseases and drug history, geographical conditions and racial differences, the absence of more patients, failure to use all inflammasome factors in the study constitute limitations.

This study is the first to determine seminal plasma and blood serum levels of NLRP3 protein in infertile men using the ELISA method in infertile men classified according to azoospermia and varicocele status. We believe that it will determine the future infertility treatment approaches and success, and many additional studies will be needed over time.

## Figures and Tables

**Table:** Statistical comparison of patients’ biochemical values in serum and semen.

		Group I						Group III						p	
***Serum***															
IL-1 beta (pg/mL)	Mean ± SD	14.2	±	5.4^2^,^3^,^4^		±	5.5	19.0	±	5.6		±	10.3	***0.041***	K
NLRP3 (pg/mL)	Mean ± SD	4.1	±	1.0^4^		±	1.2	4.2	±	1.2		±	1.3	***0.022***	K
TOS (mmol/L)	Mean ± SD	10.5	±	1.1^2^,^3^,^4^		±	2.3	13.3	±	2.0		±	2.9	***<0.001***	K
TAS (mmol/L)	Mean ± SD	1.31	±	0.12^2^,^3^,^4^		±	0.17	0.96	±	0.17		±	0.14	***<0.001***	K
OSI (AU)	Mean ± SD	0.81	±	0.11^2^,^3^,^4^		±	0.31	1.42	±	0.31		±	0.47	***<0.001***	K
***Sperm***															
IL-1 beta (pg/mL)	Mean ± SD	24.7	±	5.6^2^,^3^,^4^	35.9	±	10.2	31.9	±	9.1	40.7	±	13,5	***<0.001***	K
NLRP3 (pg/mL)	Mean ± SD	6.2	±	2.4^4^	8.1	±	3.0	6.4	±	1.8	9.8	±	3.5	***0.002***	K
TOS (mmol/L)	Mean ± SD	9.2	±	1.4^2^,^3^,^4^	13.3	±	4.3	13.5	±	4.8	17.2	±	3.2	***<0.001***	K
TAS mmol/L)	Mean ± SD	1.38	±	0.19^2^,^3^,^4^	0.98	±	0.19	0.89	±	0.39	0.88	±	0.19	***<0.001***	K
OSI (AU)	Mean ± SD	0.68	±	0.16^2^,^3^,^4^	1.38	±	0.45	2.00	±	1.58	2.08	±	0.67	***<0.001***	K
Group I Azoospermia (−) & Varicocele (−)/Group II Azoospermia (−) & Varicocele (+)															
Gruop III Azoospermia (+) & Varicocele (−)/Gruop IV Azoospermia (+) & Varicocele (+)															

K Kruskal–Wallis

2 Difference with Group II p <0.05)

3 Difference with Group III p <0.05)

4 Difference with Group IV p <0.05).

SD: standard deviation
